# Assistierter Suizid in den Medien – eine narrative Übersicht

**DOI:** 10.1007/s00103-026-04192-z

**Published:** 2026-02-13

**Authors:** Emilia Gögl, Sebastian Scherr, Markus Schäfer, Georg Fiedler, Frank Schwab

**Affiliations:** 1 Fakultät für Humanwissenschaften, Lehrstuhl Medienpsychologie, Julius-Maximilians-Universität Würzburg, Oswald-Külpe-Weg 82, 97074 Würzburg, Deutschland; 2 Philosophisch-Sozialwissenschaftliche Fakultät, Chair of Digital Health Communication, Universität Augsburg, Augsburg, Deutschland; 3 Center for Interdisciplinary Health Research, Universität Augsburg, Augsburg, Deutschland; 4 Fakultät Kultur, Medien, Psychologie, Hochschule Macromedia, Frankfurt, Deutschland; 5 Deutsche Akademie für Suizidprävention e. V. (DASP), Kassel, Deutschland

**Keywords:** Assistierter Suizid, Medien, Inhaltsanalysen, Nachahmungseffekte (Werther-Effekt), Öffentliche Kommunikation, Assisted suicide, Media, Content analyses, Imitation effects (Werther Effect), Public communication

## Abstract

Die mediale Darstellung assistierter Suizide und deren Auswirkungen auf die Rezipierenden sind bislang nur unzureichend untersucht worden. Dieser Artikel bietet einen ersten Überblick über internationale Forschungsarbeiten zu Nachrichtenbeiträgen, Filmen, Fernsehsendungen und sozialen Medien, die sich mit diesem Thema befassen. In der Fachliteratur dominieren qualitative Inhaltsanalysen, während empirische Studien zu den Auswirkungen von Darstellungen assistierten Suizids weitgehend fehlen. Die mediale Auseinandersetzung mit der Thematik bewegt sich im Spannungsfeld zwischen individueller Selbstbestimmung, gesellschaftlicher Verantwortung und möglicher normativer Einflussnahme durch öffentliche Kommunikation. Trotz medienethischer Richtlinien wird das Thema häufig emotionalisiert und anhand individueller Fälle dargestellt, die oftmals eine Befürwortung der Suizidassistenz nahelegen. Teilweise wird dadurch vermittelt, dass ein Leben mit Leiden nicht lebenswert sei. Außerdem werden Alternativen zur Suizidassistenz – wie eine umfassende Palliativversorgung – nur selten thematisiert. Es gibt Hinweise, dass Medienberichte Nachahmungseffekte auslösen können. Aufgrund der begrenzten Anzahl an Forschungsarbeiten und methodischer Unzulänglichkeiten ist es derzeit nicht möglich, weitreichende Schlussfolgerungen zu ziehen. Insbesondere im Hinblick auf potenzielle Medienwirkungen gewinnt die gründliche Untersuchung der medialen Berichterstattung und Inszenierung von assistiertem Suizid jedoch eine erhebliche gesellschaftliche Relevanz.

## Abstract (en)

Media portrayals of assisted suicide and their impact on recipients have so far received insufficient scholarly attention. This article provides an initial overview of international research on news coverage, films, television, and social media that deal with this topic. Qualitative content analyses dominate the academic literature, while empirical studies on the effects of portrayals of assisted suicide are largely absent. Media coverage of this topic navigates the tension between individual autonomy, social responsibility, and the potential normative influence of public communication. Despite media guidelines, the topic is often emotionalized and presented on the basis of individual cases, many of which implicitly advocate for assisted suicide. This sometimes conveys the message that a life with suffering is not worth living. Furthermore, alternatives to assisted suicide—such as comprehensive palliative care—are rarely discussed. There is evidence suggesting that media reports on assisted suicide can trigger imitation effects. Due to the limited number of studies and methodological shortcomings, it is currently not possible to draw far-reaching conclusions. However, particularly with regard to potential media effects, systematic research on the coverage and portrayal of assisted suicide is of critical importance.

## Einleitung

**Abb. 1:**
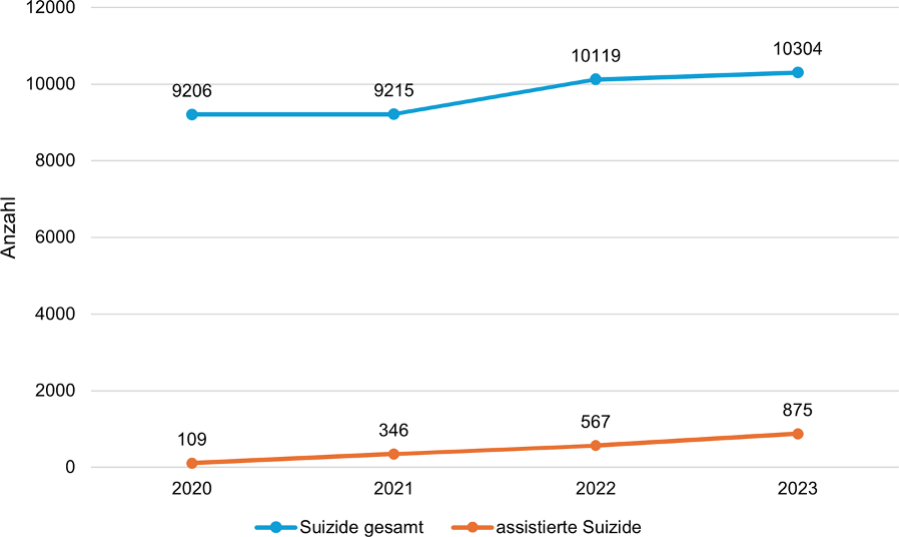
Anzahl der Suizide gesamt und der assistierten Suizide in Deutschland (2020–2023). (Datenquellen: [3, 4])

Der assistierte Suizid ist in Deutschland seit 2020 für den Assistierenden explizit straffrei [1], Initiativen für eine gesetzliche Regulierung scheiterten 2023 im Bundestag [2]. Aktuell besteht in Deutschland damit der weltweit freizügigste Zugang zur Durchführung der Suizidassistenz. Trotzdem werden assistierte Suizide in Deutschland nicht explizit erfasst, sondern vom Statistischen Bundesamt lediglich als Suizide klassifiziert. Die verfügbaren Daten der Organisationen, die in Deutschland assistierte Suizide begleiten, zeigen allerdings einen deutlichen Anstieg assistierter Suizide seit Beschluss der Straffreiheit 2020 [3], bei einem gleichzeitigen Anstieg der Gesamtzahl der Suizide ([4]; Abb. 1). Für die Medienforschung stellt sich die Frage, wie Medien mit diesem Thema umgehen und welche potenziellen Wirkungen sich aus der Berichterstattung, der Inszenierung oder dem Diskurs in klassischen und sozialen Medien für Einzelpersonen und für die Gesellschaft ergeben können.

Seit Jahrzehnten leben Menschen in Mediengesellschaften, weltweit fast überall. Medien wie Zeitungen und Zeitschriften, Bücher, Radio, Kino, Fernsehen und Internet unterhalten uns und tragen zur Information und Meinungsbildung bei. Auch das Thema Suizid ist Gegenstand medialer Aufbereitung. Bei einer sensiblen Aufbereitung mit einem Fokus auf der Bewältigung suizidaler Krisen lassen sich suizidpräventive Effekte erkennen (*Papageno-Effekt*). Dagegen kann eine unbedachte Darstellung zu einem Anstieg der Suizidzahlen führen (*Werther-Effekt;* [5]). Bei der medialen Auseinandersetzung mit assistiertem Suizid besteht ferner die Gefahr eines medial vermittelten sozialnormativen Erwartungsdrucks, der bspw. bei älteren Menschen oder Menschen mit Behinderung aufgebaut werden könnte.

Um Journalisten und Medienschaffenden den Umgang mit den Themen Suizid und Suizidassistenz zu erleichtern, veröffentlichen Netzwerke wie das Kriseninterventionszentrum in Wien, Österreich oder das Nationale Suizidpräventionsprogramm (NaSPro) in Deutschland evidenzinformierte Empfehlungen zur Darstellung von Suizid und assistiertem Suizid in fiktionalen und nonfiktionalen Medien [6–8]. Beispielsweise sollten Suizide nicht sensationalisiert, Begriffe wie „Selbstmord“ vermieden und die Privatsphäre von Suizidenten und deren Familien respektiert werden. Ziel dieser Empfehlungen ist eine verantwortungsvolle Darstellung von Suiziden in den Medien, die öffentliches Bewusstsein schafft und zur Risikominimierung und Prävention beitragen kann, ohne gleichzeitig Tabuisierung oder Stigmatisierung zu fördern.

Solche Empfehlungen zur Darstellung assistierter Suizide in den Medien erscheinen vielversprechend, da Erfahrungen mit entsprechenden Empfehlungen zu nichtassistierten Suiziden positive Ergebnisse gezeigt haben. Allerdings ist die Evidenzbasis aktuell noch unzureichend. Ziel dieser narrativen Übersicht ist es, einen Überblick über die Forschungslage zur Auseinandersetzung klassischer und sozialer Medien mit dem Thema assistierter Suizid zu geben. Hierzu werden die existierenden Befunde aus wenigen und sehr unterschiedlichen Forschungsarbeiten zusammengetragen und besprochen. Final bieten wir eine kritische Würdigung der gegenwärtigen Befundlage an.

## Allgemeine mediale Auseinandersetzung mit der Thematik assistierter Suizid

### Berichterstattung und Diskurse

In einer 2013 erschienenen niederländischen Inhaltsanalyse untersuchten Rietjens et al. [9] anhand 284 niederländischer Zeitungsartikel, ob der Begriff Euthanasie^1^ korrekt verwendet wurde und welche Argumente für und gegen Euthanasie vorgebracht wurden. Es zeigte sich, dass rund ein Viertel der Artikel mit dem Begriff etwas beschrieben, das laut dem niederländischen Gesetz keine Euthanasie ist, sondern z. B. assistierter Suizid oder die Beendigung lebensverlängernder Maßnahmen. Da Zeitungsartikel häufig als Quelle für Gesundheitsinformationen benutzt werden, ist dieses Ergebnis problematisch.

In 134 der analysierten Artikel war Euthanasie das Hauptthema. Hier wurde der Euthanasie-Begriff im Kontext verschiedener Patientengruppen wie etwa todkranker, alter oder psychisch kranker Patienten verwendet. In 94 Artikeln wurden Argumente für oder gegen Euthanasie vorgebracht. 67 % der Artikel enthielten mindestens ein Argument für Euthanasie, 74 % mindestens eins dagegen. 45 % enthielten Argumente für beide Seiten. Insgesamt zeigte sich eine Vielfalt an Pro- und Kontraargumenten. Auf der Pro-Seite waren dies v. a. die Selbstbestimmung der Patienten, die Linderung von Leiden und die Chance eines „guten Todes“. Auf der Kontraseite wurde darauf hingewiesen, dass statt der Euthanasie-Praktik eine bessere Palliativversorgung gefördert werden sollte und verletzliche Gruppen (wie alte Menschen oder Menschen mit Behinderung) geschützt werden müssen. Außerdem wurden die Schwierigkeiten einer Regulierung und die mögliche Belastung für ausführendes Fachpersonal betont. Insgesamt zeigte sich auch, dass tendenziell eher kontroverse Fälle behandelt wurden. Hier weisen die Autoren auf die Gefahr einer Verzerrung der öffentlichen Wahrnehmung der Euthanasie-Praxis hin [9].

Für eine andere niederländische Inhaltsanalyse von Van Gorp et al. [11] wurden 2700 Textfragmente aus bspw. Zeitungs- und Zeitschriftenartikel, Blogs, Tweets und Grundsatzdokumenten zur Palliativversorgung und Euthanasie aus den Jahren 2016 bis 2018 untersucht. Die Analyse orientierte sich am Konzept der Frames. Ein Frame ist in der medialen Berichterstattung ein Deutungsrahmen, der die Wahrnehmung und Interpretation eines Themas durch das Publikum lenkt [12]. Er legt fest, welche Aspekte eines Sachverhalts hervorgehoben oder vernachlässigt werden und wie das Thema moralisch oder politisch bewertet wird.

Es konnten 7 Frames und zugehörige Gegen-Frames in den untersuchten Texten identifiziert werden, von denen 2 Palliativversorgung und 5 Euthanasie entweder problematisieren oder deproblematisieren (Tab. 1; [11]). In den untersuchten Texten wurden Frames auch kombiniert, die sich gegenseitig bestärkten, bspw.: „Du sollst nicht töten“, „Dammbruchargument“, „mangelnde Willenskraft“ und „Ich bin nicht Gott“. Euthanasie-Gegner nutzten v. a. „Du sollst nicht töten“ und das „Dammbruchargument“, Befürworter v. a. „absolute Autonomie“ und „Gnade“. Menschen, die für sich Euthanasie verlangten, nutzten v. a. „Triumph der Vernunft“ sowie „Prävention“, Stakeholder aus der Palliativversorgung v. a. „Lebensqualität“.

**Tab. 1:** Problematisierende und deproblematisierende Frames von Palliativversorgung und Euthanasie in der Untersuchung von Van Gorp et al. (2021). (Quelle: [11], Lizenz: Creative Commons Attribution 4.0 International [33])

	*Problematisierender Frame*	*Deproblematisierender Frame*
*Palliativversorgung*	Angst zu sterben	Lebensqualität
	Schwere Last	Vollendung
*Euthanasie*	Du sollst nicht töten	Gnade
	Dammbruchargument	Prävention
	Mangelnde Willenskraft	Triumph der Vernunft
	Ich bin nicht Gott	Absolute Autonomie
	Medizinischer Prozess	Ökonomisches Nutzen-Denken

Die Tabelle wurde aus dem Englischen ins Deutsche übersetzt, die linke Spalte mit den Begriffen „Palliativversorgung“ und „Euthanasie“ wurden zur besseren Übersichtlichkeit ergänzt und die beiden Spalten mit Definitionen wurden entfernt

Eine deutsche Inhaltsanalyse von Menke und Kinnebrock [13], die 2016 veröffentlicht wurde, betrachtete 1130 deutschsprachige Aussagen diverser Akteure in Artikeln zur Sterbehilfe- und Pflegedebatte im Jahr 2014. Ziel war es, herauszufinden, wie Betroffene in dieser Berichterstattung repräsentiert werden und welche Konzepte von Würde in diesen Druckmedien vorhanden sind. Es zeigte sich, dass Betroffene in der Berichterstattung zwar häufig thematisiert wurden, allerdings seltener selbst zu Wort kamen als bspw. Journalisten, Politiker oder Ärzte. Wenn sie zu Wort kamen, äußerten sie sich v. a. zur eigenen Situation oder sprachen über Personen aus dem Umfeld wie Ärzte, Pflegepersonal und Angehörige. Aussagen von Betroffenen zu Sterbehilfe und Pflege allgemein oder zu zugehörigen rechtlichen Belangen waren kaum zu finden.

Die Autoren unterschieden in ihrer Analyse 2 Würdekonzepte: das Würdekonzept der Autonomie und das der Verbundenheit. In 321 der 1130 analysierten Aussagen wurde eines der beiden Würdekonzepte thematisiert. Autonomie wurde dabei 167-mal, Verbundenheit 154-mal als Grundlage von Würde verstanden. Insbesondere Journalisten und Sterbehelfer bezogen sich in ihrem Verständnis von Würde auf Autonomie, Politiker bezogen sich etwas häufiger auf Verbundenheit. An der Pflege und Fürsorge beteiligte Personen (Ärzte, Pflegepersonal, Angehörige) verstanden Würde deutlich häufiger im Sinne der Verbundenheit als der Autonomie. Sterbewillige selbst definierten Würde in den Artikeln ausschließlich über Autonomie.

Auch in einer 2009 veröffentlichten Inhaltsanalyse von Hahnen et al. [14] wurde der Würdebegriff thematisiert. Dazu wurden 433 deutsche Zeitungsartikel aus den Jahren 2006 und 2007 zur Thematik Sterbehilfe analysiert. Der Begriff „Würde“ wurde in den untersuchten Artikeln 160-mal verwendet. Er wurde dabei sowohl von Befürwortern als auch Gegnern der Sterbehilfe verwendet. Dabei wurde auf einer Seite Selbstbestimmung, auf der anderen Fürsorge als Würde verstanden. In den untersuchten Artikeln waren sich beide Seiten darin einig, dass die Würde durch eine Verlängerung des Leidens gefährdet wird. Außerdem wurden Selbstbestimmung (auch im Kontext der Patientenverfügung) und die Gefahr der Unterversorgung thematisiert. Es zeigte sich auch, dass Unsicherheiten bezüglich des Begriffs Sterbehilfe herrschten und er in verschiedenen Bedeutungen verwendet wurde, wodurch teilweise Falschaussagen entstanden.

Haller und Ralph [15] beschäftigten sich in ihrer Analyse mit Frames in 375 US-amerikanischen und 117 britischen Zeitungsartikeln aus den Jahren 1996 bis 1998. Genauer wurden die darin enthaltenen Glaubenssätze über Menschen mit Behinderung in der Berichterstattung über ärztlich assistierten Suizid untersucht. Dabei zeigte sich zunächst, dass die Problematik für Menschen mit Behinderung im Kontext ärztlich assistierten Suizids kaum behandelt wurde. Weiter konnten die Autoren 6 Frames in den analysierten Artikeln identifizieren: Erstens wurde der bekannte Advokat für ärztlich assistierten Suizid, Dr. Jack Kevorkian, in den Vordergrund gerückt. Die der Debatte zugrunde liegende Frage wurde als „für oder gegen Kevorkian“ dargestellt. Zweitens waren Personen aus Kevorkians Umfeld – wie seine Anwälte – zentrale Quellen, wenn es darum ging, ärztlich assistierten Suizid zu erklären. Drittens wurde ärztlich assistierter Suizid nicht als Frage des Menschenrechts für Menschen mit Behinderung behandelt, sondern v. a. als Frage von legalen, religiösen und moralischen Unsicherheiten. Viertens wurden Behinderungen in den analysierten Artikeln allgemein in die Kategorie tödlicher Krankheiten gesteckt. Fünftens wurde eine Botschaft von „besser tot als behindert“ übermittelt. Sechstens wurde argumentiert, dass heutige assistierte Suizide nicht mit der vergangenen Euthanasie von Menschen mit Behinderung in bspw. der Zeit des Nationalsozialismus zu vergleichen seien.

Pollock und Yulis [16] analysierten im Rahmen eines „community structure approach“ 288 US-amerikanische Zeitungsartikel zum ärztlich assistierten Suizid zwischen Januar 1993 und Januar 1997. Der Ansatz geht davon aus, dass die Art und Weise, wie Medien Themen aufgreifen und darstellen, systematisch mit den strukturellen Eigenschaften einer Gemeinschaft (Community) zusammenhängt (z. B. Größe einer Stadt, Bildungsniveau, sozioökonomische Merkmale). Korrelationsanalysen, Faktorenanalysen und Regressionen lieferten folgende Erkenntnisse: Nur im Westen zeigten Artikel eine überwiegend befürwortende Haltung gegenüber assistiertem Suizid, Zeitungen mit einer ablehnenden Ausrichtung lagen an oder in der Nähe der Ostküste. Die jeweilige Ausrichtung zeigte deutliche Zusammenhänge mit Merkmalen der Städte, in denen die untersuchten Zeitungen veröffentlicht wurden. Eine Rolle spielten „Zugang“ (guter Zugang zu Medien und Gesundheitsversorgung), „Privileg“ (hohes Einkommen, viele Computernutzer, gute Bildung), „Verletzlichkeit“ (hohe Arbeitslosigkeit, hohe Armut, wenig junge Menschen) und „Alter“ (viele Einwohner über 75 Jahren). Zugang und Privileg korrelierten jeweils signifikant mit einer befürwortenden Berichterstattung, Verletzlichkeit und Alter mit einer ablehnenden Berichterstattung. Die Faktoren Alter und Zugang klärten 46,3 % der Varianz auf.

### Film und Fernsehen: Fiktionale und nonfiktionale Inszenierungen

In einer 2017 erschienenen Analyse untersuchte Schmidt [17] 26 internationale Filme und Serienepisoden zum Thema assistierter Suizid. Es zeigte sich, dass die untersuchten Bewegtbildinhalte meist auch zu Entertainment-Zwecken dienten (der Autor benutzt in diesem Zusammenhang den Begriff „Sterbetainment“). So wurde die Thematik des assistierten Suizids v. a. in Form von Beziehungsdramen behandelt. Die assistierenden Personen in den Geschichten waren häufig nahestehende Personen und medizinisches Fachpersonal zugleich und standen damit vor dem Dilemma zwischen ihrem berufsbedingten Ethikverständnis und ihrer Verpflichtung gegenüber der geliebten Person. Das Beziehungsdrama zeigte sich auch in der Verkündung des Sterbewunschs (häufig bei einem gemeinsamen Essen) und den beidseitigen Überzeugungsversuchen für bzw. gegen die Suizidassistenz. Die sterbewillige Person stellte die Suizidassistenz dabei als ultimativen Liebesbeweis dar.

Die Sterbewilligen in den Geschichten befanden sich teilweise in einem ambivalenten Spannungsfeld zwischen dem Wunsch nach einem Ende des Leids auf der einen und dem Wunsch nach einem Weiterleben auf der anderen Seite. Der Autor identifizierte dabei eine Entwicklung über die Jahrzehnte bezüglich des Ursprungs des Wunsches nach Suizidassistenz: Grund war nicht länger nur die Vermeidung von Leid, sondern auch die Vermeidung der als würdelos empfundenen Abhängigkeit. Zudem wurden diverse Krankheitsbilder (neben tödlichen Krebserkrankungen bspw. auch Querschnittslähmungen, weniger jedoch „unästhetische“ Krankheiten wie entstellende Krebserkrankungen) und unterschiedliche Altersgruppen bei den Sterbewilligen inszeniert. Thematisiert wurde auch die Frage, ob psychische Erkrankungen eine Suizidassistenz „verdienen“ oder ob sie körperlich Erkrankten vorbehalten bleibt. Die Möglichkeit der Palliativversorgung fand dagegen in den untersuchten Formaten wenig Beachtung.

Tendenziell folgten Patienten nur bedingt den Vorschlägen des behandelnden Fachpersonals und bestimmten selbst über den weiteren Verlauf ihres Lebens. Insgesamt stand die Selbstbestimmung der Patienten in den untersuchten Bewegtbildinhalten häufig über der ärztlichen Autorität.

Die Geschichten endeten – wie es dramaturgisch nahe liegt – mit dem Tod der sterbewilligen Person. Selten wurden die Folgen für Angehörige über den Tod der sterbewilligen Person hinaus beleuchtet.

Neuner et al. [18, 19] setzten sich in 2 Texten kritisch mit der Fernsehdokumentation „Right to Die“ auseinander. Sie wurde 2008 im Vereinigten Königreich ausgestrahlt und behandelte den vom Schweizer Verein „Dignitas“ assistierten Suizid eines Mannes mit amyotropher Lateralsklerose. Die Autoren legten nahe, dass diese und ähnliche Berichterstattungen über assistierte Suizide einen Werther-Effekt hervorrufen könnten. Besonders für vulnerable Menschen könnte die Darstellung des assistierten Suizids als friedliche, angemessene und akzeptable Lösung in Hinblick auf eine Nachahmungstendenz bedenklich sein. Die Autoren appellierten daher an eine verantwortungsvolle Berichterstattung.

Ein prominentes deutsches TV-Beispiel zum Thema Suizidassistenz ist das Fernsehstück „GOTT“ aus dem Jahr 2020, das auf dem gleichnamigen Theaterstück von Ferdinand von Schirach basiert. Darin wird der Antrag des 78-jährigen Richard Gärtner auf Suizidassistenz in einem fiktiven Ethikrat verhandelt [20]. Die Ausstrahlung löste eine breite mediale Debatte über Selbstbestimmung, Würde und ärztliche Verantwortung aus und zeigte, wie Fernsehen zur gesellschaftlichen Meinungsbildung beitragen kann. Die Erstausstrahlung wurde von 3,88 Mio. Zuschauerinnen und Zuschauern verfolgt, was 4,6 % der Bevölkerung entsprach. An der anschließenden Abstimmung beteiligten sich 546.000 Personen (ca. 14 % der Zuschauenden bzw. 0,65 % der Gesamtbevölkerung); dabei votierten rund 71 % für die Genehmigung der Suizidassistenz für Gärtner [21]. Zwar liegen kritische Pro- und Kontratexte zu diesem Medienexperiment vor (didaktische, interpretative und theologisch bzw. ethische Reflexionen), es fehlen jedoch empirische, methodisch belastbare Arbeiten.

## Digitale Medien und soziale Netzwerke über assistierten Suizid

In einer Diskursanalyse von 3568 Texten (bspw. Blogs, Threads in sozialen Medien oder Website-Artikel) aus einer 2017 in Neuseeland stattfindenden Euthanasie-Debatte untersuchten Jaye et al. [22], wie Menschen an der Debatte teilnehmen und welche Meinungen sie vertreten. Basierend auf der Anzahl der Postings ist anzunehmen, dass das Thema 2017 von gesellschaftlichem Interesse war. Die Autoren erkannten 3 Hauptthemen, die in den Postings behandelt wurden: erstens die Frage, wie die Debatte um Suizidassistenz aussehen sollte. In dieser Kategorie der Postings wurde die Validität der Debatte infrage gestellt und soziale Einflussfaktoren auf die Debatte diskutiert. Es ging auch um den Inhalt der Debatte und die Frage, welchen Autoritäten es zusteht, zur Debatte beizutragen. Zweitens die Frage, wie ein mögliches Suizidassistenzgesetz aussehen sollte, wozu es dienen würde und wie es zu einem Gesetzesbeschluss kommen könnte. Hier wurden in den Postings Kriterien für den Anspruch auf Suizidassistenz sowie Tötungsmethoden und Rahmenbedingungen der Suizidassistenz diskutiert. Weiter wurden politische Implikationen thematisiert und für eine Mehrheitsentscheidung argumentiert. Außerdem wurde diskutiert, wie der aktuelle Status quo in Neuseeland aussieht und welche versteckten Motive (wie Sparen von Kosten für das Gesundheitssystem) es hinter einer Legalisierung geben könnte. Drittens wurden individuelle und soziale Konsequenzen einer Legalisierung diskutiert. Als mögliche individuelle Konsequenzen wurden hier ein unfreiwilliger oder vorzeitiger Tod, aber auch ein „besserer“ Tod genannt. Im sozialen Kontext wurden gesellschaftliche Werte und praktische Implikationen thematisiert, ebenso wie die öffentliche Gesundheit.

Insgesamt zeigte sich in den Postings, dass die Meinungen zu dem Thema nicht als „entweder oder“ zu beschreiben waren, sondern sich sehr komplex darstellten.

Eine weitere Inhaltsanalyse im Bereich Suizidassistenz und Social Media wurde von Scourfield et al. [23] durchgeführt. Mithilfe geografischer Filter untersuchten sie britische Tweets zum Zeitpunkt der Ausstrahlung einer Seifenoper-Episode im britischen Fernsehen am 20.01.2014, in der ein assistierter Suizid stattfand. Dabei zeigte sich, dass die Anzahl der Tweets, die das Wort „Suizid“ am Tag der Sendung enthielten, im Vergleich zum Rest der betrachteten Woche statistisch signifikant erhöht war. Dies galt allerdings nicht für die Anzahl der Tweets, deren Inhalt auf eine Suizidabsicht schließen ließ. Es gab also keine Anzeichen für eine verstärkte Kommunikation möglicher Suizidabsichten zum Zeitpunkt der Sendung auf Twitter, lediglich das Thema war präsenter.

Die Autoren untersuchten weiter den Inhalt der Tweets, die am Abend der Ausstrahlung veröffentlicht wurden und das Wort „Suizid“ enthielten (*N* = 798), und ordneten sie verschiedenen Kategorien zu. Insgesamt enthielten 196 Tweets Informationen oder unterstützende Worte. Moralische Fragen im Zusammenhang mit Suizid wurden in 146 Beiträgen behandelt. Über den Suizid einer Person (exkl. Suizidattentate) berichteten 128 Tweets. Weitere 112 Tweets enthielten Posts, die von den Autoren als „leichtfertige Hinweise auf Suizid“ klassifiziert wurden. Lediglich 22 Beiträge wiesen auf konkrete Suizidabsichten hin, und 10 Tweets enthielten Gedenken oder Beileidsbekundungen. Weitere 184 Tweets ließen sich keiner dieser sechs Hauptkategorien zuordnen. Die Autoren sahen in Twitter eine Art öffentlichen Resonanzraum („civic reactive forum“).

## Fallorientierte Berichterstattung zu assistierten Suiziden

### Familienassistierte Suizide (FAS) in Kanada und Großbritannien

In einer 2009 veröffentlichten Analyse 25 kanadischer Print-Nachrichtenberichte zum Fall von Marielle Houle, die angeklagt wurde, ihrem an multipler Sklerose (MS) erkrankten Sohn Charles Fariala 2004 beim Suizid geholfen zu haben, untersuchten Schwartz und Lutfiyya [24], wie die Medien über den Fall berichteten und wie sie Behinderung darstellten.

Houle wurde in den Medien als liebende Mutter dargestellt – im Kontext der Tat wurde sie als mutige, mitfühlende Märtyrerin beschrieben. Im Kontext des anschließenden Gerichtsprozesses charakterisierten die analysierten Berichte sie als verloren, verletzlich und fragil. Insgesamt ergab sich eher das Bild einer heldenhaften, liebevollen Mutter als das einer Täterin.

Fariala wurde als zurückgezogen und depressiv beschrieben. Außerdem zeichneten die Medien das Bild eines ehemals aktiven, stolzen jungen Mannes, der seit der MS-Erkrankung an Schmerzen litt, Einschränkungen hatte und einen Kontrollverlust erlebte. Die Krankheit selbst wurde als abnormal dargestellt, ein Leben mit ihr als wertlos und ausschließlich negativ. Es ergab sich daher das Bild, dass der Tod dem Weiterleben mit fortschreitender Erkrankung vorzuziehen sei. Dass Fariala an Depressionen gelitten haben könnte, wurde nicht thematisiert.

Gesetze zur Suizidassistenz wurden als veraltet beschrieben, die Notwendigkeit eines Wandels betont – auch durch Referenzen zu ähnlichen Fällen. Anhand individueller Fälle wurden die Vorteile der Suizidassistenz dargestellt. Es wurde nicht zwischen verwandten Begriffen, wie Euthanasie und Suizidassistenz, unterschieden. Die Medienberichterstattung positionierte sich durchweg pro Legalisierung des assistierten Suizids.

Birenbaum-Carmeli et al. [25] analysierten 29 britische Zeitungsartikel aus den Jahren 1996 bis 2000, die 3 Fälle des familienassistierten Suizids (FAS) behandelten. In allen 3 Fällen halfen die Täter einem unheilbar kranken Familienmitglied dabei, sich das Leben zu nehmen. Die Suizidenten hatten mehrmals den Wunsch nach Suizidassistenz geäußert, alle Täter bekannten sich vor Gericht schuldig. Die verstorbenen Personen wurden als willensstark, mutig, unabhängig und heldenhaft, der Suizid als Befreiung aus der Krankheit dargestellt. Bei diesen Beschreibungen beriefen sich Journalisten auf Aussagen der Täter.

Die Täter stellten in den Artikeln die Schlüsselfiguren dar. Sie wurden als würdevoll, moralisch, fürsorglich und gefangen in unglücklichen Umständen beschrieben, die Suizidassistenz selbst als höchstes Zeichen der Liebe und des Mitgefühls. Da die Täter sicher waren, das Richtige für ihre erkrankten Familienmitglieder getan zu haben, kamen in den Artikeln weder Bedenken noch Reue der Täter zum Ausdruck. Die Beziehungen der verstorbenen Personen zu ihren Familienmitgliedern wurden als liebevoll, harmonisch und fürsorglich charakterisiert. Der FAS selbst wurde als Moment der Verbundenheit beschrieben. Außerdem wurde bezüglich des FAS stets Konsens unter den Familienmitgliedern berichtet.

Das Justizsystem wurde als veraltet inszeniert, Richter selbst hingegen als mitfühlend und durch das Aussprechen milder Urteile auf der Seite der Täter und Verstorbenen stehend. Die Fälle wurden durch die Medien als Ausnahmen charakterisiert. Insgesamt zeigte sich die Berichterstattung wohlwollend gegenüber den FAS-Fällen. Kritische Stimmen wurden gar nicht oder nur knapp erwähnt.

In einer weiteren 2007 veröffentlichten Analyse derselben 29 Zeitungsartikel zu den 3 FAS-Fällen schlussfolgerten Banerjee und Birenbaum-Carmeli [26], dass der FAS in den Artikeln als geordneter und rationaler Akt dargestellt und die eigentliche Komplexität dahinter durch mehrere Faktoren kaschiert wurde. Erstens wurden Gesundheit und Autonomie als Norm behandelt, während das degenerative Sterben als abnormal und Verlust von Würde dargestellt wurde. Zweitens wurde der Entscheidungsprozess der Täter, ihren Familienmitgliedern beim Suizid zu assistieren, als eindeutig, ohne Zweifel oder Reue beschrieben und durch diese Reduzierung den Autoren nach beinahe trivialisiert. Drittens wurden ethische Fragen hinter dem FAS nicht gesamtgesellschaftlich erörtert, sondern nur auf die Einzelfälle bezogen – hierbei wurden Täter stets als moralisch und die Fälle und ihre Umstände als außergewöhnlich beschrieben. Viertens wurden vorangegangene Fehlversuche des FAS beschönigt. So reichten bei 2 der 3 Fälle die Medikamente nicht aus und die Täter mussten auf Ersticken zurückgreifen, um den FAS zu Ende zu bringen. Damit einhergehende Herausforderungen ethischer oder emotionaler Natur wurden kaum berichtet.

Zuletzt wurde in den Artikeln die rechtliche Lage als einziges tatsächliches Hindernis dargestellt.

### Psychisch erkrankte Euthanasie-Patientin in Belgien

De Hert et al. [27] analysierten 789 flämische Nachrichtenbeiträge, die zwischen dem 01.12.2019 und dem 01.03.2020 veröffentlicht wurden und den ersten Kriminalfall im Kontext Euthanasie für psychisch kranke Patienten in Belgien behandelten. Eine 38-jährige Belgierin mit Borderline-Persönlichkeitsstörung und/oder Autismus nahm im Jahr 2010 Euthanasie in Anspruch. Nachdem die Familie der Patientin geklagt hatte, weil die strengen Voraussetzungen für Euthanasie angeblich nicht erfüllt gewesen waren, ging der Fall 2020 vor Gericht. Die Inhaltsanalyse untersuchte, wie über den Fall berichtet wurde und ob sich Unterschiede zwischen verschiedenen Zeitungen erkennen lassen.

Die Patientin wurde überwiegend als tragische Figur dargestellt, die behandelnde Psychiaterin einerseits als manipulative Person, die zu schnell Euthanasie genehmigte, andererseits als verantwortungsvolle Person, die versucht, Menschen zu retten. Der Euthanasie-ausführende Arzt wurde als nervös und unbeholfen dargestellt, die Euthanasie als chaotisch. Der dritte beratende Arzt wurde als guter Mann charakterisiert, der nicht über die Abläufe der Euthanasie aufgeklärt wurde. Die Familie der Patientin wurde als dysfunktional, traumatisiert und ihre Fürsorge und Aufmerksamkeit der Patientin gegenüber als unzureichend beschrieben.

In den analysierten Artikeln wurde kritisiert, dass nicht alle Behandlungsoptionen vor der Euthanasie ausgeschöpft wurden und der Entscheidungsprozess bei der Kontrollkommission nicht ordentlich ablief. Außerdem wurde spekuliert, ob die Kirche einen Einfluss auf den Fall gehabt haben könnte. Die Zeitungen behandelten v. a. rechtliche, persönliche/familiäre und kontroverse Aspekte des Falls.

Der Tonfall der Überschriften war überwiegend neutral gegenüber dem Fall (47 %), gefolgt von negativ (42 %) und positiv (11 %). Insgesamt enthielten 90 % der Überschriften und 89 % der Artikel keine wesentlichen Standpunkte zu dem Fall oder waren neutral gehalten.

### US-Amerikanerin Brittany Maynard

Auch die mediale Darstellung des Falls der US-Amerikanerin Brittany Maynard, die 2014 aufgrund eines fortgeschrittenen unheilbaren Hirntumors in den US-Bundesstaat Oregon umzog, um dort Suizidassistenz zu beanspruchen, fand wissenschaftliche Beachtung. In einer Inhaltsanalyse untersuchten Lauffer und Baker [28] 203 Elemente US-amerikanischer Berichterstattung (bspw. Nachrichten, Leserbriefe und Editorials), die zwischen dem 09.10.2014 und dem 09.12.2014 veröffentlicht wurden. Ziel der Analyse war es, herauszufinden, wie Maynards Fall dargestellt wurde und welche ideologischen Vorstellungen über Suizidassistenz durch die Berichte über ihren Fall gefördert wurden.

Es zeigten sich in der Berichterstattung vor allem 3 Frames. Der erste Frame stellte Maynard als „tragische Figur“ dar, als Opfer des Tumors. Ihr junges Alter wurde betont, ebenso der Kontrast vor vs. nach der Diagnose – Vorher-Bilder stellten Maynard als fröhliche, attraktive junge Frau dar, während die Nachher-Fotos sie in einem erschöpften, von Krankheit gezeichneten Zustand zeigten. Leserbriefen lagen v. a. christliche Ideologien zugrunde, die Maynard das Recht auf den Tod verweigerten. In Meinungsbeiträgen hingegen wurde Maynard eher als mutig oder würdevoll dargestellt und das Mitgefühl mit ihr betont.

Der zweite Frame stellte den assistierten Suizid als „friedlichen Tod“ dar, der ein gutes und ruhiges Ende des Leidens markiert. Betont wurden hierbei die Symptome, die Maynards Lebensqualität reduzierten, und dass sie eigentlich nicht sterben, sondern nur einem furchtbaren Tod entkommen wolle.

Der dritte Frame drehte sich um Maynard als „sympathische Advokatin für die Recht-zu-sterben-Bewegung“. Maynard selbst stellte ihre Entscheidung für die Suizidassistenz als eine Möglichkeit dar, Kontrolle zu behalten und eine Wahl zu haben, im Vergleich zu einem schmerzhaften Tod durch ihren Tumor. Ihr Verständnis von Würde wurde durch einige Medienberichte infrage gestellt. Ebenso kritisiert wurden die Zelebrierung ihrer Entscheidung, das mediale Ausschlachten ihres Todes sowie Euphemismen für Suizidassistenz, wie bspw. „Tod in Würde“.

Die Inhaltsanalyse zeigte in Bezug auf vorherrschende ideologische Glaubenssätze zu assistiertem Suizid, dass diese stabil und negativ waren – nur in Oregon, Washington und Vermont (wo Suizidassistenz zu dem Zeitpunkt legalisiert war) waren sie stabil und positiv.

Einige Aspekte von Maynards Geschichte wurden gestützt durch amerikanische Ideologien wie Individualismus (ihr Wunsch, selbst über ihren Tod zu bestimmen) und den Wert der Jugendlichkeit. Argumente, die gegen die Suizidassistenz vorgebracht wurden, waren etwa die Möglichkeit des sozialen und finanziellen Drucks auf kranke oder alte Menschen, der unfreiwillige Entscheidungen für die Suizidassistenz zur Folge haben könnte. Auch religiöse Ideologien und das Argument, dass Mediziner heilen, nicht töten sollten, konnten in der Berichterstattung beobachtet werden. Ebenso wurde argumentiert, dass die Freiheit des Individuums durch die Zugehörigkeit zu einer Gesellschaft grundsätzlich in gewisser Weise eingeschränkt ist. Die Berichterstattung zeigte sich insgesamt häufiger positiv gegenüber Suizidassistenz (51,7 %) als negativ (20,2 %) oder neutral (28,1 %).

## Hinweise auf Auswirkungen der medialen Auseinandersetzung mit assistiertem Suizid

Die Gruppe „Minnesotans Against Assisted Suicide“ veröffentlichte 2015 einen Bericht, in dem sie auch den Fall Brittany Maynard behandelte [29]. Sie kritisierte den Umgang der Medien mit dem Fall, da die Berichterstattung nicht die Richtlinien der Weltgesundheitsorganisation (WHO) für Berichterstattung über Suizide befolgte.

Außerdem analysierte sie Daten der Oregon Health Authority, einer Regierungsbehörde im US-Bundesstaat Oregon, die Hinweise auf einen Werther-Effekt lieferten. So stieg zum Zeitpunkt der intensiven Berichterstattung über Maynard im Oktober 2014 die Zahl der tödlichen Verschreibungen auf insgesamt 18 – ein Anstieg um 39,4 % im Vergleich zum Monatsdurchschnitt von 2014. Die Anzahl der tatsächlichen Todesfälle durch assistierten Suizid lag im gleichen Monat bei 12 und stieg im November, dem Todesmonat von Maynard, sogar auf 15 an. Damit lag die Anzahl der tatsächlichen Todesfälle im November 71,4 % höher als der Monatsdurchschnitt von 2014. In diesem Monat ereigneten sich zudem mehr assistierte Suizide als in den anderen Monaten der Jahre 2010–2014.

Weitere Hinweise auf einen möglichen Werther-Effekt liefert eine 2003 erschienene Publikation von Frei et al. [30]. Die Autoren untersuchten, ob die intensive Berichterstattung in der Schweizer Basel-Region über den EXIT-Doppelsuizid des lokal berühmten Paares G. einen Einfluss auf die Fallzahlen der durch EXIT assistierten Suizide hatte. Dafür wurden 2 Zeitintervalle verglichen: der Zeitraum zwischen dem 20.03.1993 und dem 19.03.1995 (I1) und der Zeitraum zwischen dem 20.03.1995 und dem 19.03.1997 (I2). Der Doppelsuizid des Paares G. wurde am 19.03.1995 bekannt. Es sei erwähnt, dass die Berichterstattung gegen die Richtlinien des Center for Disease Control zur verantwortungsvollen Berichterstattung über Suizide verstieß.

Im I1 fanden 7 EXIT-assistierte Suizide in der Basel-Region statt, im I2 waren es 28, was einen signifikanten Anstieg von I1 zu I2 bedeutet. Zehn der 28 Suizidenten im I2 litten nicht an einer unheilbaren oder ernsthaften Krankheit. Folglich wurde eine ausschließliche Erklärung des Anstiegs in I2 mit medizinischen Umständen ausgeschlossen. Schon die Zahlen allein weisen auf einen möglichen Werther-Effekt hin. Hinzu kommt, dass die Autoren darüber berichten, dass ein ehemaliger EXIT-Manager und Sterbehelfer angab, dass eine Frau explizit den Doppelsuizid des Paares G. erwähnte, als sie sich mit dem Wunsch nach Suizidassistenz an EXIT wandte.

## Assistierter Suizid in den Medien – Stand, Desiderata und Ausblick

Die mediale Darstellung von assistiertem Suizid bewegt sich in einem Spannungsfeld zwischen individueller Selbstbestimmung, gesellschaftlicher Verantwortung und möglicher normativer Einflussnahme durch öffentliche Kommunikation. Entgegen medienethischen Richtlinien emotionalisieren Medien das Thema Suizidassistenz häufig anhand individueller Fälle. Diese werden dabei oft so inszeniert, dass eine Befürwortung von Suizidassistenz überwiegt – dieser Eindruck entsteht zumindest durch Inhaltsanalysen zu konkreten Fällen.

Dabei lassen einige der untersuchten Medienprodukte Gefahrenpotenziale erkennen: Teilweise wird die Botschaft, ein Leben mit Behinderungen oder Leid sei nicht lebenswert, vermittelt. Alternativen wie eine gute Palliativversorgung werden zu selten behandelt. Betroffene werden als mutige Helden glorifiziert, die Thematik Suizidassistenz unterkomplex behandelt und dadurch als Lösung für körperliches Leid teilweise normalisiert. In Teilen der Literatur konnten sogar Hinweise auf einen Werther-Effekt bei Berichterstattung über Suizidassistenz beobachtet werden [29, 30].

Insgesamt zeigte sich auch, dass die mediale Thematisierung von assistiertem Suizid bislang ein wenig beforschtes Feld ist. Eine Analyse entlang der in der Medienforschung etablierten Lasswell-Formel („Who says what in which channel to whom with what effect?“) verdeutlicht, dass zwar einige Aspekte (z. B. „what“ und „channel“) bereits in Form von meist qualitativen Inhaltsanalysen adressiert wurden, jedoch empirisch belastbare Wirkungsforschung fehlt und auch Rezipierendenperspektiven (z. B. bzgl. Alter, Vulnerabilität, Bildung) bislang weitgehend unbeachtet bleiben (Tab. 2).

**Tab. 2:** Übersicht nach Lasswell-Formel – Forschungsstand zur medialen Thematisierung assistierten Suizids

*Komponente*	*Beschreibung*	*Bisherige Forschung*	*Forschungsbedarf (Desiderata)*
*Who (Kommunikatoren)*	Journalisten, Autoren, Produzenten, Aktivisten	Kaum untersucht	Systematische Untersuchung der Zielsetzungen und redaktionellen Routinen
*Says what (Inhalt)*	Darstellung des assistierten Suizids in Print, TV, Film, Social Media	Vielfältige, meist qualitative Inhaltsanalysen	Quantitativ repräsentative Inhaltsanalysen
*In which channel (Medium)*	Printmedien, Fernsehen, Film, Social Media	Gut dokumentiert für traditionelle Medien	Neue Medienformate (TikTok, YouTube etc.) bislang wenig untersucht
*To whom (Rezipierende)*	Allgemeinbevölkerung, Betroffene, Angehörige, vulnerable Gruppen	Praktisch nicht erforscht	Rezeptionsstudien, auch mit Fokus auf Vulnerabilität
*With what effect (Wirkung)*	Nachahmungseffekte, Normen und Einstellungen	Vereinzelte Hinweise (z. B. EXIT-Fall)	Wirkungsforschung: experimentell, longitudinal, surveybasiert

Die wenigen vorliegenden Studien zu Wirkungsrisiken [29, 30] deuten auf mögliche Nachahmungseffekte hin, lassen aber aufgrund methodischer Limitationen kaum generalisierbare Aussagen zu. Problematisch für entsprechende Forschung in Deutschland ist auch, dass hierzulande keine offizielle Datenerfassung zu assistierten Suiziden stattfindet – sie werden vom Statistischen Bundesamt lediglich als Suizide klassifiziert. Auch Medienmonitoring-Strukturen könnten die Forschungsbemühungen in diesem Bereich unterstützen.

Erforscht werden sollte ebenfalls, weshalb Medienschaffende den Empfehlungen für eine verantwortungsvolle Berichterstattung über Suizidfälle – basierend auf den betrachteten Analysen – selten zu folgen scheinen und wie dies geändert werden könnte, besonders, da Hinweise auf die Effektivität entsprechender Interventionen vorliegen [31, 32].

Mit Blick auf eine evidenzbasierte Medienpolitik und potenzielle suizidpräventive Maßnahmen ist dies ein bedeutsames Forschungsfeld. Ein verantwortungsvoller gesellschaftlicher Diskurs zu assistiertem Suizid kann nur auf Grundlage valider Daten gelingen. Eine systematische Forschungsagenda ist notwendig – sowohl zur Analyse bestehender Darstellungen als auch zu ihrer Wirkung in einem digitalen und mediatisierten öffentlichen Raum. Hierzu bedarf es (auch interdisziplinärer) Bemühungen aus u. a. Medienforschung, Psychologie, Ethik und Gesundheitswissenschaften.

## Footnotes

1 Euthanasie ist in den Niederlanden die aktive und beabsichtigte vorzeitige Beendigung eines Lebens durch einen Arzt auf den ausdrücklichen Wunsch des Patienten hin [10]. Damit die Euthanasie straffrei bleibt, müssen strenge Sorgfaltskriterien erfüllt werden. Es besteht zudem eine Meldepflicht an eine Kontrollkommission.
